# Consensus guidelines for botulinum toxin therapy: general algorithms and dosing tables for dystonia and spasticity

**DOI:** 10.1007/s00702-021-02312-4

**Published:** 2021-02-26

**Authors:** Dirk Dressler, Maria Concetta Altavista, Eckart Altenmueller, Roongroj Bhidayasiri, Saeed Bohlega, Pedro Chana, Tae Mo Chung, Carlo Colosimo, Klemens Fheodoroff, Pedro J. Garcia-Ruiz, Beomseok Jeon, Lingjing Jin, Petr Kanovsky, Ivan Milanov, Federico Micheli, Olga Orlova, Sanjay Pandey, Zvezdan Pirtosek, Maja Relja, Raymond Rosales, José Alberto Sagástegui-Rodríguez, Gholam Ali Shahidi, Sofia Timerbaeva, Xinhua Wan, Uwe Walter, Fereshte Adib Saberi

**Affiliations:** 1 Movement Disorders Section, Department of Neurology, Hannover Medical School, Carl-Neuberg-Str. 1, 30625 Hannover, Germany; 2 Department of Neurology, A.C.O. San Filippo Neri, Rome, Italy; 3 Institute of Music Physiology and Musicians’ Medicine, Hanover University of Music, Drama and Media, Hannover, Germany; 4 Chulalongkorn Centre for Excellence on Parkinson’s Disease and Related Disorders, King Chulalongkorn Memorial Hospital, Bangkok, Thailand; 5 Department of Neurology, King Faisal Specialist Hospital, Riyyad, Kingdom of Saudi Arabia; 6 Department of Neurology, University de Santiago de Chile, Santiago de Chile, Chile; 7 University of Sao Paulo, Sao Paulo, Brazil; 8 Department of Neurology, Santa Maria University Hospital, Terni, Italy; 9 Gailtal-Klinik, Hermagor, Austria; 10 Department of Neurology, Fundacion Jimenez Diaz, Madrid, Spain; 11 Department of Neurology, Seoul National University, Seoul, Republic of Korea; 12 Department of Neurology, Tongji University School of Medicine, Shanghai, China; 13 Department of Neurology, Palacky University, Olomouc, Czech Republic; 14 Department of Neurology, Medical University of Sofia, Sofia, Bulgaria; 15 Department of Neurology, Hospital de Clínicas José de San Martín, University of Buenos Aires, Buenos Aires, Argentina; 16 Clinic ‘Cecil Plus’, Moscow, Russia; 17 Department of Neurology, Govind Ballabh Pant Institute of Postgraduate Medical Education and Research, New Delhi, India; 18 Department of Neurology, Ljubljana University, Ljubljana, Slovenia; 19 Department of Neurology, University of Zagreb, Zagreb, Croatia; 20 Department of Neurology, University of Santo Tomas, Manila, Philippines; 21 Department of Neurology, University of Monterrey, Monterrey, Nueva Leon Mexico; 22 Department of Neurology, Iran University of Medical Sciences, Tehran, Iran; 23 Scientific Research Institute of Neurology, Moscow, Russia; 24 Department of Neurology, Peking Union Medical College, Beijing, China; 25 Department of Neurology, Rostock University, Rostock, Germany; 26 IAB—Interdisciplinary Working Group for Movement Disorders, Hamburg, Germany

**Keywords:** Botulinum toxin, Therapy, Consensus guidelines, Dystonia, Spasticity, Treatment algorithms, Dosing tables, Target muscles, Total dose, Typical dose, Dose limits, Dose variability

## Abstract

Botulinum toxin (BT) therapy is a complex and highly individualised therapy defined by treatment algorithms and injection schemes describing its target muscles and their dosing. Various consensus guidelines have tried to standardise and to improve BT therapy. We wanted to update and improve consensus guidelines by: (1) Acknowledging recent advances of treatment algorithms. (2) Basing dosing tables on statistical analyses of real-life treatment data of 1831 BT injections in 36 different target muscles in 420 dystonia patients and 1593 BT injections in 31 different target muscles in 240 spasticity patients. (3) Providing more detailed dosing data including typical doses, dose variabilities, and dosing limits. (4) Including total doses and target muscle selections for typical clinical entities thus adapting dosing to different aetiologies and pathophysiologies. (5) In addition, providing a brief and concise review of the clinical entity treated together with general principles of its BT therapy. For this, we collaborated with IAB—Interdisciplinary Working Group for Movement Disorders which invited an international panel of experts for the support.

## Introduction

Botulinum toxin (BT) therapy is a complex and highly individualised therapy defined by treatment algorithms and injection schemes. The treatment algorithms consist of the set of parameters describing BT therapy and the ways they are combined and modified to adapt them to the individual patient’s treatment situation. The injection scheme describes the individual patient’s target muscles and their dosing.

Consensus guidelines have tried to standardise and to improve BT therapy. There have been several attempts to develop such guidelines and to make them publicly available. The most widespread ones have been produced by We Move Inc, New York City, NY, USA for dystonia, spasticity, cerebral palsy, and BT type B some 15 years ago and were based on original work by Brin (1997). More recent guidelines cover dystonia only (Albanese et al. 2011, 2015), whilst another one also covers spasticity and several other indications (Simpson et al. 2016). However, two of them (Albanese et al. 2011; Simpson et al. 2016) are presenting treatment algorithms only and do not include dosing tables. The dosing table included in the third guideline (Albanese et al. 2015) covers cervical dystonia only and—due to a very heterogenous database—recommends BT doses varying by factors from two to six for individual muscles, thus reducing their practical usefulness considerably.

We wanted to update and improve consensus guidelines by: (1) acknowledging recent advances of treatment algorithms (2) basing dosing tables on statistical analyses of real-life treatment data originating from a reference centre with a minimum of legal and economic restrictions to perform BT therapy; (3) providing more detailed dosing data, including typical doses, dose variabilities, and dosing limits for all relevant target muscles; (4) including total doses and target muscle selections for typical dystonia (cervical dystonia, facial dystonia, oromandibular dystonia, arm dystonia, and axial dystonia) and spasticity indications (arm spasticity, leg spasticity, hemispasticity, paraspasticity, and tetraspasticity) thus adapting dosing to different aetiologies (spasticity and dystonia) and pathophysiologies (task-specific dystonia and non-task-specific dystonia); (5) in addition, providing a brief and concise review of the clinical entity treated together with general principles of its BT therapy.

This project was organised by IAB—Interdisciplinary Working Group for Movement Disorders, a German organisation with a worldwide reach to promote interdisciplinary collaboration for improving the understanding and therapy of movement disorders. IAB invited an international panel of experts with an outstanding reputation for BT therapy to provide their input.

## Methods

### Definitions

The following definitions are used:

**Table:** 

Treatment algorithms	The set of parameters used to describe a BT therapy and the ways they are combined and modified to adapt it to the individual patient’s treatment situation
Injection scheme	Describes the individual patient’s target muscles and their dosing
Dosing table	Describes BT doses for target muscles
Real-life treatment data	Treatment data derived from the actual use of BT therapy
Target muscle	A muscle selected to receive BT applications
Total dose	The amount of BT applied to a patient at one injection series
Interinjection interval	The interval between two subsequent injection series
Therapeutic window	The sensitivity of a target muscle to receive BT without showing functional impairment
Dystonia ratio	The amount of dystonic muscle activity in relation to the target muscle’s maximal voluntary activity
Drug potency labelling	The potency of a BT drug as described by the manufacturer
Drug stability	The potency changes of the unreconstituted or reconstituted BT drug over time
Guidance techniques	Techniques including ultrasound and EMG (with or without electric stimulation) to control the application of BT
Dosing variability	Indicated by the standard deviation of the BT doses applied to a target muscle as documented in the reference centre’s database
Dosing limits	Indicated by the minimum and maximum of the BT doses applied to a target muscle as documented in the reference centre’s database
Typical dose	Indicated by the mean BT dose applied to a target muscle as documented in the reference centre’s database

### Design

These consensus guidelines are based on a panel review of current treatment algorithms and statistical analysis of real-life treatment data deriving from a specialised reference centre.

### Reference centre

The reference centre is the Movement Disorders Section, Department of Neurology, Hannover Medical School, Hannover, Germany. It was founded 12 years ago by one of the authors (DD) and is specialised in BT therapy. Currently, the centre’s annual BT usage is in excess of 20,000 100 MU vials of onabotulinumtoxinA (ONA, Botox^®^, Allergan, Dublin, Ireland) and incobotulinumtoxinA (INCO, Xeomin^®^, Merz Pharmaceuticals, Frankfurt/M, Germany).

### Database

Data used for this evaluation derived from real-life data routinely collected in the computerised reference centre’s database during the last 11 years. For this study, 420 dystonia patients (59.8 ± 14.0 years, 38% males, 62% females) with cervical dystonia (*n* = 200), facial dystonia (*n* = 100), writer’s cramp (*n* = 50), oromandibular dystonia (*n* = 50), arm dystonia (*n* = 10), and axial dystonia (*n* = 10) and 240 spasticity patients (55.8 ± 15.3 years, 59% males, 41% females) with arm spasticity (*n* = 80), hemispasticity (*n* = 85), leg spasticity (*n* = 25), paraspasticity (*n* = 20), and tetraspasticity (*n* = 30) were consecutively collected until pre-set numbers of patients for each indication were reached. All patients had undergone a phase of BT therapy optimisation and had to be on a stable BT therapy regimen for at least 1.5 years. The total number of patients evaluated reflect about 30% of all dystonia patients and about 15% of all spasticity patients receiving BT therapy at this institution. Altogether, 1831 BT injections in 36 different target muscles were analysed for the treatment of dystonia and 1593 BT injections in 31 different target muscles were analysed for the treatment of spasticity.

All data storage and analysis were performed anonymised and according to the regulations applicable.

### BT drugs analysed

This evaluation is based on an analysis of BT therapy using ONA and INCO. AbobotulinumtoxinA (ABO, Dysport^®^, Ipsen, Billancourt, France) was not included in this evaluation as its potency labelling is substantially different from the potency labelling of ONA and INCO. With uncertain conversion factors ranging from 1:2 to 1:5 between the potency labelling of ONA/INCO and ABO, we found it unsafe to include ABO. RimabotulinumtoxinB, a BT-type B drug, was also not included as its therapeutic profile is principally different from BT-type A drugs.

### Treatment algorithms used by the reference centre

BT therapy at the reference centre is based on algorithms developed during the last 34 years by one of the authors (DD) and his team. The reference centre is able to perform BT therapy with a minimum of economic and legal restrictions. It is thus able to exploit BT therapy’s maximum benefit. For all patients treated, BT therapy is free of costs. Regulatory recommendations on target muscle selection and dosing, total doses, inter-injection intervals and contraindications are modified wherever necessary. Permission to perform quantitative and qualitative off-label use was generally granted. Total doses of up to INCO 1500 MU according to the concept of the ‘BT high dose therapy’ and inter-injection intervals down to 6 weeks according to the concept of the ‘BT short interval therapy’ may be applied where necessary. They will be discussed in the section ‘General treatment algorithms’. The standard dilution is 2.5 ml 0.9% NaCl/H_2_O per ONA/INCO 100 MU. The standard volume per injection site is 0.5 ml (20 MU), for facial injections 0.2 ml (4 MU). The number of injection sites is determined by the BT doses applied to each target muscle.

## General treatment algorithms

### Principle of BT therapy

The basic principle of BT therapy for motor indications is to select the appropriate target muscles and to apply appropriate BT doses to them, or in short: ‘Hit the right muscle with the right dose’. These two aspects are documented in the injection scheme. The development of the injection scheme is based on knowledge and experience, and will be highly individualised for each patient treated. It may require several subsequent injection series to be optimised.

### Target muscle selection (‘the right muscle’)

Target muscles are selected by documentation of the patient’s pathological positioning and movements. Based on the understanding of physiological muscle functioning, pathologically active muscles are identified. Muscle pain may contribute to additional information. Compensatory muscle activity and protective postures have to be identified and need to be distinguished from primary pathological muscle activity.

### Target muscle dosing (‘the right dose’)

Once a target muscle is identified, the degree of BT-induced paresis, i.e., the BT dose applied, has to be decided. Only necessary BT doses should be applied. They should be as low as possible to reduce functional impairment, BT spread into adjacent muscles, excessive total doses and unnecessary costs. However, they should be high enough to produce robust and lasting therapeutic effects. Dosing depends on the target muscle’s mass, its therapeutic window and the paresis risk in adjacent muscles. Therapeutic windows (Dressler 2000) are shown in Table 1. The dosing tables provided here describe typical doses with their variability and limits. Individual BT dosing within these limits will be higher when the pathological muscle activity is high and when supportive agonistic muscles are available. BT doses will be lower when the pathological muscle activity is functionally useful, as in spasticity’s paresis or in dystonic tremor (see Table 2). For dystonia, the degree of dystonic involvement may be calculated with the dystonia ratio (dystonic muscle activity in relation to the maximal muscle activity as measured by the surface EMG amplitude) (Dressler 2000). General dose modifiers (see Table 2) (Dressler et al. 2018) are also applicable.

**Table 1:** Therapeutic windows of different target muscles Modified from Dressler (2000)

Therapeutic window	Target muscle
Narrow	Finger extensors
	Muscles of the angle of the mouth
	Finger flexors
Medium	Neck muscles
Wide	M. orbicularis oculi

**Table 2:** Modifiers of botulinum toxin doses

	BT dose adjustment
*Specific modifiers*	
Pathological muscle activity strong	↑
Agonistic muscles available	↑
Pathological muscle activity limited	↓
Pathological muscle activity functionally useful	↓
*General modifiers*	
Muscle mass reduced old age, female sex and atrophy from previous BT injections	↓
Muscle mass increased athletic training	↑
Co-morbidity	
Myasthenia gravis Emmerson (1994), Fasano et al. (2005), Dressler (2010)	↓
Lambert–Eaton syndrome Erbguth et al. (1993)	↓
BT antibodies	↑
General BT sensitivity increased	↓

*BT* botulinum toxin

### Total BT doses

Registration documents usually recommend maximal total BT doses in the region of 300–400 MU. Recent studies introducing the BT high dose therapy, however, demonstrate the toxicological and immunological safety of maximal total INCO doses of up to 1250 MU, thus establishing the ‘BT high dose therapy’ ((Dressler et al. 2014a; Wissel et al. 2017). Note that these doses originate from an increase in the number of target muscles rather than from excessive BT dosing in individual target muscles. Note also that maximal total doses are influenced by the dilution used and the number of target muscles selected.

### Interinjection intervals

Reduced therapeutic effects at the end of the injection cycle may be compensated by increased BT doses. However, this effect is limited. Alternatively, interinjection intervals may be reduced. Originally, interinjection intervals were recommended to be not less than 12 weeks. Recent studies indicate that INCO may be applied without toxicological or immunological complications at intervals of less than 12 weeks (Dressler et al 2014a), thus establishing the ‘BT short interval therapy’. Interinjection intervals may be as short as 6 weeks (Dressler and Saberi 2017).

### BT drugs

BT drugs differ in many aspects. As biologicals, their manufacturing process influences them beyond their physical and chemical properties. With respect to therapeutic and adverse effects, BT drugs based on different BT types show considerable differences, whereas BT drugs based on the same BT type are very similar. The effects of different excipients including the use of human serum albumin, gelatine and polysorbate are controversially discussed. The lack of complexing proteins and the particular manufacturing process used in INCO has reduced antigenicity.

### Drug potency labelling

Despite governmental regulations on standardised potency measurements, the potency labelling of BT drugs is not directly comparable. The potency labelling of ONA and INCO may be compared with a conversion factor of 1:1 (Dressler et al. 2012, 2014b, 2018). Conversion factors between ONA/INCO and other BT drugs are still controversial.

### BT application

All BT-type A drugs need to be reconstituted with 0.9% NaCl/H_2_O. This generates and determines a dilution effect. Unless for special indications, e.g., treatment of hyperhidrosis, dilutions should not vary to increase patient safety. A dilution of 100 MU of ONA/INCO in 2.5 ml 0.9% NaCl/H_2_O produces volumes that are easily injectable and adequate in relation to the target muscle volume. An injection volume of 0.5 ml per injection site seems to be best suited.

### Drug stability

Unreconstituted BT drugs have very long shelf lives. Most BT drugs require temperature restrictions. Only INCO may be stored and transported at room temperature. Reconstituted BT drugs should be used within 24 h. Recently published INCO data, however, indicate a stability of at least 1 year (Dressler and Bigalke 2017). Obviously, this has considerable economic implications.

### Guidance techniques

Usually, the BT application is performed using basic anatomical techniques including palpation of the target muscle’s belly, its tendons and insertions and references to landmarks. Muscle pain and its localisation provide additional orientation. Target muscle palpation should only be performed when the target muscle is activated. For identification of deeper laying target muscles, application of some gentle pressure will become necessary. Special guidance techniques may be useful to target forearm muscles and to separate individual muscle fascicles when they are involved individually as it is typically the case in writer’s cramp. Guidance techniques may be EMG with and without electric stimulation (O’Brien 1997; Ajax et al. 1998) and ultrasound (Walter and Dressler 2018). Tomographic imaging techniques are not useful especially when they involve radiation.

## Dosing tables for BT therapy of dystonia

When the reader wants to treat a specific BT indication, he or she will find a brief review of its clinical presentation together with a general description of the principles of its BT therapy. For each target muscle, the reader will then find typical BT doses, dose variabilities and dose limits. The usage indicates the likelihood of the target muscle’s clinical involvement.

### Cervical dystonia (Table 3)

**Table 3:** Dosing table for cervical dystonia

(A) Patient age (*M* ± SD) (years)	Patient sex ratio (%)	Additional dystonia manifestations (%)	Number of target muscles (*M* ± SD) (min–max) (*n*)	Total botulinum toxin dose (*M* ± SD) (min–max) (MU)
58.5 ± 13.5	Males: 38	Facial: 23	5.7 ± 1.8	262.6 ± 141.6
	Females: 62	Oromandibular: 17	1–13	40–860
		Axial: 7		
		Arm: 6		
		Leg: 1		

(B) Target muscle	Botulinum toxin dose			Target muscle usage (% per indication)
	Typical dose (mean) (MU)	Dose variability (standard deviation (MU)	Dose limits (min–max) (MU)	
M. trapezius/Pars descendens	44.7	27.5	10–200	78
M. splenius capitis	55.2	33.2	10–300	75
M. sternocleidomastoideus	46.4	21.1	10–120	52
M. levator scapulae	34.2	15.3	10–80	31
Mm. scalenii	36.5	14.9	20–80	29
M. trapezius/Pars horizontalis	46.0	27.4	20–180	20
Deep neck muscles	32.2	15.4	20–60	3
Additional muscles	Suprahyoid muscles platysma			

Analysis of 200 consecutive patients. (A) Patient characteristics, additional dystonia manifestations, and general botulinum toxin therapy characteristics. (B) Target muscles, botulinum toxin doses and target muscle usage

*M ± SD* mean ± standard deviation

Cervical dystonia is the most common form of dystonia. In our group of 200 consecutive cervical dystonia cases, the patient age was 58.4 ± 13.5 years and the patient sex ratio 38% males and 72% females. 23% of patients with predominant cervical dystonia had additional facial dystonia, 17% oromandibular dystonia, 7% axial dystonia, 6% arm dystonia, and 1% leg dystonia. The total BT dose in cervical muscles was 262.6 ± 141.6 MU (minimum 40 MU, maximum 860 MU). The number of cervical target muscles was 5.7 ± 1.8 (minimum 1, maximum 13). Most frequently used target muscles were M. trapezius/Pars descendens (78%), M. splenius capitis (75%), M. sternocleidmastoideus (52%), M. levator scapulae (31%), Mm. scalenii (29%), M. trapezius/Pars horizontalis (20%) and the deep neck muscles (3%). Occasionally, the suprahyoid muscles and the platysma were also target muscles.

The highly visible sternocleidomastoid muscle is *not* the most frequently involved target muscle. Bilateral BT injections into the M. sternocleidomastoid are possible necessarily producing dysphagia. Throughout the literature, there is confusion about the anatomical attribution of the nuchal muscles. For historical reasons, we use M. trapezius/Pars descendens to describe the nuchal paravertebral muscles including the M. splenius cervicis and M. semispinalis capitis. The actual M. trapezius/Pars descendens is a very thin muscle rotating the head into the opposite direction—similar to the M. sternocleidomastoideus. Its force and functional relevance are negligible. Deep neck muscles describe a muscle group including M. rectus capitis posterior minor, M. rectus capitis posterior major, M. obliquus superior, and M. capitis obliquus inferior. They are strong head rotators and head extensors. Similar functionality makes selective BT injections requiring EMG or ultrasound identification unnecessary.

### Facial dystonia (Table 4)

**Table 4:** Dosing table for facial dystonia

(A) Patient age (*M* ± SD) (years)	Patient sex ratio (%)	Additional dystonia manifestations (%)	Number of target muscles (*M* ± SD) (min–max) (*n*)	Total botulinum toxin dose (*M* ± SD) (min–max) (MU)
65.2 ± 13.3	Males: 31	Cervical: 45	3.7 ± 1.8	78.8 ± 31.6
	Females: 69	Oromandibular: 25	1–10	4–220

(B) Target muscle	Botulinum toxin dose			Target muscle usage (% per indication)
	Typical dose (mean) (MU)	Dose variability (standard deviation) (MU)	Dose limits (min–max) (MU)	
M. orbicularis oculi/Pars orbitalis	32.9	9.9	8–80	91
M. procerus	6.0	3.5	2–20	32
M. orbicularis oculi/Pars palpebr	12.9	4.8	4–24	26
M. mentalis	6.2	2.6	2–10	12
M. frontalis	6.2	2.3	4–10	9
M. risorius	5.3	2.9	2–12	9
Platysma	33.0	21.0	4–80	8
M. nasolabialis	7.0	3.5	4–12	4
M. depressor anguli oris	4.8	3.3	4–8	2
M. nasalis	5.0	1.2	4–6	2
Additional muscles	M. orbicularis oris			

Analysis of 100 consecutive patients. (A) Patient characteristics, additional dystonia manifestations, and general botulinum toxin therapy characteristics. (B) Target muscles, botulinum toxin doses and target muscle usage

*M ± SD* mean ± standard deviation

Facial dystonia is the second most common form of dystonia. In our group of 100 consecutive facial dystonia patients, their age was 65.2 ± 13.3 years and the sex ratio was 31% males and 69% females. 45% of these patients had additional cervical dystonia and 25% oromandibular dystonia. The total facial BT dose was 78.8 ± 31.6 MU (minimum 4 MU, maximum 220 MU). The number of target muscles was 3.7 ± 1.8 (minimum 1, maximum 10). Most frequently used target muscles were M. orbicularis oculi/Pars orbitalis (91%), M. procerus (32%), M. orbicularis oculi/Pars palpebralis (26%), M. mentalis (12%), M. frontalis (9%), M. risorius (9%), Platysma (8%), M. nasolabialis (4%), M. depressor anguli oris (2%) and M. nasalis (2%). Occasionally, the M. orbicularis oris may be target muscle.

BT injections into perioral muscles are prone to produce paretic adverse effects. The M. frontalis should be used carefully as it is an auxiliary eyelid opening muscle. The M. orbicularis oculi/Pars palpebralis is used when there is a component of eyelid opening apraxia.

### Writer’s cramp (Table 5)

**Table 5:** Dosing table for writer’s cramp

(A) Patient age (*M* ± SD) (years)	Patient sex ratio (%)	Additional dystonia manifestations (%)	Number of target muscles (*M* ± SD) (min–max) (*n*)	Total botulinum toxin dose (*M* ± SD) (min–max) (MU)
61.6 ± 20.8	Males: 60	Cervical: 2	2.5 ± 1.5	70.3 ± 55.3
	Females: 40		1–6	8–230

(B) Target muscle	Botulinum toxin dose			Target muscle usage (% per indication)
	Typical dose (mean) (MU)	Dose variability (standard deviation) (MU)	Dose limits (min–max) (MU)	
M. flexor digitorum superficialis	21.8	13.9	8–70	48
M. flexor carpi ulnaris	32.7	18.2	10–80	42
M. extensor carpi ulnaris	35.4	13.1	10–60	34
M. extensor carpi radialis	28.0	13.2	10–50	30
M. flexor digitorum profundus	19.9	10.3	8–40	30
M. flexor policis longus	22.7	13.5	6–60	28
M. flexor carpi radialis	13.0	5.5	8–20	12
M. pronator teres	47.0	39.3	8–100	8
M. extensor indicis	34.3	43.8	10–100	8
M. extensor policis	9.3	1.2	8–10	6
Additional muscles	M. extensor digitorum			
	M. flexor indicis			
	M. supinator			
	M. deltoideus			
	M. trapezius/Pars descendens			
	M. triceps brachii			

Analysis of 50 consecutive patients. (A) Patient characteristics, additional dystonia manifestations, and general botulinum toxin therapy characteristics. (B) Target muscles, botulinum toxin doses and target muscle usage

*M ± SD* mean ± standard deviation

Writer’s cramp is another common manifestation of dystonia. Unlike most other dystonias, it is task-specific, i.e., it only occurs when the specific motor program of writing is executed. In our group of 50 consecutive writer’s cramp patients their age was 61.6 ± 20.8 years and the sex ratio was 60% males and 40% females. 2% of these patients had additional cervical dystonia. The total arm BT dose was 70.3 ± 55.3 MU (minimum 8 MU, maximum 230 MU). The number of target muscles in the arm was 2.5 ± 1.5 (minimum 1, maximum 6). Most frequently used target muscles were M. flexor digitorum superficialis (48%), M. flexor carpi ulnaris (42%), M. extensor carpi ulnaris (34%), M. extensor carpi radialis (30%), M. flexor digitorum profundus (30%), M. flexor pollicis longus (28%), M. flexor carpi radialis (12%), M. pronator teres (8%), M. extensor indicis (8%), and M. extensor pollicis (6%). Occasionally, the M. extensor digitorum, M. flexor indicis, M. supinator, M. deltoideus, M. trapezius/Pars horizontalis and the M. triceps brachii may be target muscles.

BT application in writer’s cramps frequently requires guidance either by ultrasound or by electromyography with or without electrostimulation. BT dosing in writer’s cramp is highly individual including a large number of potential target muscles in a wide range of BT doses. BT should be dosed carefully to avoid paretic adverse effect easily occurring because of the narrow therapeutic window of the target muscles.

### Oromandibular dystonia (Table 6)

**Table 6:** Dosing table for oromandibular dystonia

(A) Patient age (*M* ± SD) (years)	Patient sex ratio (%)	Additional dystonia manifestations (%)	Number of target muscles (*M* ± SD) (min–max) (*n*)	Total botulinum toxin dose (*M* ± SD) (min–max) (MU)
57.9 ± 14.6	Males: 40	Cervical: 38	3.68 ± 1.7	127.5 ± 69.9
	Females: 60	Facial: 30	2–8	40–280
		Arm dystonia: 10		
		Axial dystonia: 3		

(B) Target muscle	Botulinum toxin dose			Target muscle usage (% per indication)
	Typical dose (mean) (MU)	Dose variability (standard deviation) (MU)	Dose limits (min–max) (MU)	
M. masseter	36.7	14.0	20–60	97
M. pterygoidei	31.6	13.2	8–60	44
M. temporalis	42.0	23.9	20–80	24
M. submandibularis	16.0	5.5	10–20	13
Platysma	33.3	11.5	20–40	8

Analysis of 50 consecutive patients. (A) Patient characteristics, additional dystonia manifestations, and general botulinum toxin therapy characteristics. (B) Target muscles botulinum toxin doses and target muscle usage

*M ± SD* mean ± standard deviation

 In our group of 40 consecutive oromandibular dystonia patients, their age was 57.9 ± 14.6 years and the sex ratio was 40% males and 60% females. 38% of these patients had additional cervical dystonia, 30% facial dystonia, 10% arm dystonia and 3% axial dystonia. The total oromandibular BT dose was 127.5 ± 69.9 MU (minimum 40 MU and maximum 280 MU). The number of target muscles was 3.7 ± 1.7 (minimum 2, maximum 8). Most frequently used target muscles were M. masseter (97%), Mm. pterygoidei (44%), M. temporalis (24%), M. submandibularis (13%) and Platysma (8%). Occasionally, the M. risorius and the M. mentalis may be target muscles.

The Mm. pterygoidei can easily be injected through the incisura mandibulae. Electromyography requiring thick combination needles seems unnecessary as dystonic involvement is usually affecting both, the lateral and the medial pterygoid muscles.

### Arm dystonia (Table 7)

**Table 7:** Dosing table for arm dystonia

(A) Patient age (*M* ± SD) (years)	Patient sex ratio (%)	Additional dystonia manifestations (%)	Number of target muscles (*M* ± SD) (min–max) (*n*)	Total botulinum toxin dose (*M* ± SD) (min–max) (MU)
37.2 ± 19.7	Males: 40	Cervical: 80	3.8 ± 2.3	156.0 ± 143.3
	Females: 60	Oromandibular: 10	1–10	40–540

(B) Target muscle	Botulinum toxin dose			Target muscle usage (% per indication)
	Typical dose (mean) (MU)	Dose variability (standard deviation) (MU)	Dose limits (min–max) (MU)	
M. deltoideus	40.0	16.3	20–60	70
M. flexor carpi ulnaris	40.0	16.3	20–60	70
M. pectoralis	40.0	24.5	20–80	50
M. brachioradialis	36.0	16.7	20–60	50
M. biceps brachii	40.0	16.3	20–60	40
M. flexor carpi radialis	40.0	16.3	20–60	40
M. pronator	46.7	11.5	40–60	30
Mm. latissimus dorsi/teres maior	53.3	23.1	40–80	30

Analysis of 10 consecutive patients. (A) Patient characteristics, additional dystonia manifestations, and general botulinum toxin therapy characteristics. (B) Target muscles, botulinum toxin doses and target muscle usage

*M ± SD* mean ± standard deviation

Non-task-specific arm dystonia is a less common manifestation of dystonia. In our group of 10 consecutive arm dystonia patients, their age was 37.2 ± 19.7 years and thus considerably lower than the age of other focal dystonias. The sex ratio was 40% males and 60% females. Its isolated occurrence is very rare. 80% of these patients had additional cervical dystonia and 10% oromandibular dystonia. Total arm BT dose was 156.0 ± 143.3 MU (minimum 40 MU, maximum 540 MU). The number of target muscles was 3.8 ± 2.3 (minimum 1, maximum 10). Most frequently used target muscles were M. deltoideus (70%), M. flexor carpi ulnaris (70%), M. pectoralis (50%), M. brachioradialis (50%), M. flexor carpi radialis (40%), M. biceps brachii (40%), M. pronator (30%) and M. latissimus dorsi/M. teres maior (30%).

### Axial dystonia (Table 8)

**Table 8:** Dosing table for axial dystonia

Patient age (mean ± SD) (years)	Patient sex ratio (%)	Additional dystonia manifestations (%)	Number of segmental levels (mean ± SD) (*n*)	Botulinum toxin dose per level and side (min–max) (MU)	Total botulinum toxin dose (mean ± SD) (min–max) (MU)
61.7 ± 11.6	Males: 30	Cervical: 50	3.3 ± 1.8	40–60	218.0 ± 97.3
	Females: 70	Oromandibular: 10			80–400

Analysis of 10 consecutive patients. Patient characteristics, additional dystonia manifestations, and general botulinum toxin therapy characteristics and botulinum toxin doses

*M ± SD *mean ± standard deviation

Axial dystonia is another less frequent manifestation of dystonia. In our group of 10 consecutive axial dystonia patients, their age was 61.7 ± 11.6 years and the sex ratio was 30% males and 70% females. Axial dystonia usually occurs together with other dystonia manifestations. In 50% of our patients, it is cervical dystonia, in 10% facial dystonia. The total axial BT dose was 218.0 ± 97.3 MU (minimum 80 MU, maximum 800 MU). The number of segmental levels injected was 3.3 ± 1.8. The dose per segmental level on one side was 40–60 MU.

### Leg dystonia

Isolated leg dystonia is very rare. It almost only occurs in widespread dystonia. BT doses are similar to those used in spasticity.

### Wide-spread dystonia

Wide-spread dystonia includes all patients with dystonia exceeding two adjacent focal dystonias, i.e., segmental dystonia with more than two localisations, with hemidystonia and with generalised dystonia. BT therapy consists of treatment of the focal dystonic elements. New treatment algorithms allowing high-dose application offer improved treatment options.

## Dosing tables for BT therapy of spasticity

### General comments

Treatment algorithms for spasticity are similar to those of dystonia. BT dosing, however, differs: the principle difference between spasticity and dystonia is the obligatory presence of paresis in spasticity. This means that functional improvement in spasticity is less pronounced than in dystonia, thus changing the treatment goals in spasticity more towards pain reduction, prevention of contractures and facilitation of physiotherapeutic training programs. BT doses for spasticity tend to be higher than those for dystonia, as paretic adverse effects are a lesser concern and robust antispastic effects are more often required. In principle, BT therapy of dystonia rarely involves leg muscles. Except for writer’s cramp, arm muscles are also rarely involved. If they are involved in dystonia, their involvement is usually proximal, whereas it is usually distal in writer’s cramp. In spasticity, the typical pattern of arm muscle involvement includes shoulder abduction or adduction, elbow flexion, pronation, wrist flexion, finger flexion and thumb flexion. The typical pattern in leg muscles includes hip adduction, knee extension, and equinovarus position of the foot. Facial, cervical, and axial muscles are only rarely involved. Mandibular muscles may be involved and their involvement should be examined on a routine basis.

### Arm spasticity (Table 9)

**Table 9:** Dosing table for arm spasticity

(A) Patient age (*M* ± SD) (years)	Patient sex ratio (%)	Additional target muscles (%)	Number of target muscles (*M* ± SD) (*n*)	Total botulinum toxin dose (*M* ± SD) (MU)
59.1 ± 14.5	Males: 65	M. trapezius/Pars descendens: 8	Average: 6.5 ± 2.7	Average: 386.8 ± 167.2
	Females: 35	M. levator scapulae: 4	Minimum: 1	Minimum: 60
		M. splenius capitis: 3	Maximum: 12	Maximum: 900

(B) Target muscle	Botulinum toxin dose			Target muscle usage (% per subtype)
	Typical dose (mean) (MU)	Dose variability (standard deviation) (MU)	Dose limits (min–max) (MU)	
M. pectoralis	58.5	24.1	40–120	50
Mm. latissimus dorsi/teres maior	64.4	21.0	40–120	34
M. deltoideus	58.6	16.6	40–80	18
M. biceps brachii	63.5	23.4	20–140	79
M. brachioradialis	43.7	12.4	20–80	34
M. triceps brachii	53.3	19.3	40–100	30
M. brachialis	45.7	9.8	40–60	9
M. flexor carpi ulnaris	61.7	21.4	40–100	74
M. flexor carpi radialis	52.5	20.3	40–100	30
M. pronator teres	42.0	6.2	40–60	25
M. extensor carpi ulnaris	65.0	30.0	40–100	5
M. extensor carpi radialis	70.0	42.4	40–100	3
M. flexor digitorum superficialis	74.9	31.7	40–140	88
M. flexor digitorum profundus	75.7	32.5	10–150	76
M. flexor pollicis longus	39.4	10.8	20–60	40
M. extensor digitorum	70.0	14.1	60–80	3
Thumb clench	47.3	18.0	20–80	28
Mm. lumbricales	45.6	9.2	40–60	23

Analysis of 80 consecutive patients. (A) Patient characteristics, additional target muscles, and general botulinum toxin therapy characteristics. (B) Target muscles, botulinum toxin doses and target muscle usage

*M ± SD* mean ± standard deviation

 Arm spasticity is the largest group of patients treated for spasticity. In our group of 80 consecutive arm spasticity cases, the patient age was 59.1 ± 14.5 years and the patient sex ratio 65% males and 35% females. 8% of patients with arm spasticity also received BT therapy of the M. trapezius/Pars descendens, 4% of the M. levator scapulae, and 3% of the M. splenius capitis. The total BT dose in arm muscles was 386.8 ± 167.2 MU (minimum 60 MU, maximum 900 MU). The number of arm target muscles treated was 6.5 ± 2.7 (minimum 1, maximum 12). Most frequently used target muscles were arm flexors including M. flexor digitorum superficialis (88%), M. biceps brachii (79%), M. flexor digitorum profundus (76%), and M. flexor carpi ulnaris (74%). The total BT dose in arm spasticity was more than double the total BT dose in arm dystonia and more than 5 times the total BT dose in writer’s cramp. Finger extensors are particularly sensitive to the BT application. Treatment of shoulder muscles may reduce pain considerably, especially on a long-term perspective.

### Hemispasticity (Table 10)

**Table 10:** Dosing tables for hemispasticity

(A) Patient age (*M* ± SD) (years)	Patient sex ratio (%)	Additional target muscles (%)	Number of target muscles (*M* ± SD) (*n*)	Total botulinum toxin dose (*M* ± SD) (MU)
58.6 ± 14.7	Males: 60	M. masseter: 2	Average: 7.8 ± 3.3	Average: 495.2 ± 189.4
	Females: 40	M. trapezius/Pars horizontalis: 5	Minimum: 2	Minimum: 80
			Maximum: 16	Maximum: 900

(B) Target muscle	Botulinum toxin dose			Target muscle usage (% per subtype)
	Typical dose (mean) (MU)	Dose variability (standard deviation) (MU)	Dose limits (min–max) (MU)	
M. pectoralis	59.4	20.6	40–100	42
Mm. latissimus dorsi/teres maior	60.0	23.1	20–100	33
M. deltoideus	56.9	25.6	20–100	15
M. biceps brachii	62.6	21.1	20–100	68
M. brachioradialis	44.8	15.4	20–80	25
M. triceps brachii	54.3	14.5	20–80	16
M. brachialis	50.0	15.4	40–80	7
M. flexor carpi ulnaris	60.8	21.7	20–100	59
M. flexor carpi radialis	52.7	24.3	20–100	26
M. extensor carpi ulnaris	20.0	0	20	2
M. extensor carpi radialis	40.0	28.3	20–60	2
M. flexor digitorum superficialis	78.4	24.8	40–140	75
M. flexor digitorum profundus	78.8	25.8	40–150	67
M. flexor pollicis longus	43.2	10.0	20–60	22
M. pronator teres	35.6	11.0	40–60	18
Thumb clench	40.0	7.8	20–60	16
Mm. lumbricales	50.0	19.4	60–80	12
Mm. adductors	80.0	26.2	40–120	9
M. quadriceps femoris	80.0	40.6	40–200	20
Hamstrings	112.9	56.9	40–260	18
M. gastrocnemius/Caput mediale	72.4	51.5	20–220	54
M. gastrocnemius/Caput laterale	43.5	14.3	20–80	27
M. soleus	49.1	21.0	20–100	25
M. tibialis posterior	70.0	25.7	20–140	52
M. tibialis anterior	70.0	38.3	40	10
M. flexor digitorum brevis	76.8	33.4	30–200	26
M. flexor digitorum longus	64.7	11.5	60–80	4
M. extensor hallucis longus	52.7	20.5	40–100	13
M. flexor hallucis longus	64.4	16.7	40–100	11

Analysis of 85 consecutive patients. (A) Patient characteristics, additional target muscles, and general botulinum toxin therapy characteristics. (B) Target muscles, botulinum toxin doses and target muscle usage

*M ± SD* mean ± standard deviation

 Hemispasticity is the second largest group of patients treated for spasticity. In our group of 85 consecutive hemispasticity cases, the patient age was 58.3 ± 14.8 years and the patient sex ratio 60% males and 40% females. The total BT dose in arm and leg muscles was 495.2 ± 189.4 MU (minimum 80 MU, maximum 900 MU). The number of arm and leg target muscles treated was 7.8 ± 3.3 (minimum 2, maximum 16). Most frequently used target muscles were M. biceps brachii (80%), M. pectoralis (77%), M. flexor carpi ulnaris (53%) and M. flexor digitorum profundus (53%).

### Leg spasticity (Table 11)

**Table 11:** Dosing table for leg spasticity

(A) Patient age (*M* ± SD) (years)	Patient sex ratio (%)	Additional target muscles (%)	Number of target muscles (*M* ± SD) (*n*)	Total botulinum toxin dose (*M* ± SD) (MU)
53.7 ± 14.2	Males: 32	None	Average: 4.3 ± 1.4	Average: 270.4 ± 95.7
	Females: 68		Minimum: 1	Minimum: 40
			Maximum: 7	Maximum: 400

(B) Target muscle	Botulinum toxin dose			Target muscle usage (% per subtype)
	Typical dose (mean) (MU)	Dose variability (standard deviation) (MU)	Dose limits (min–max) (MU)	
M. iliopsoas	50.0	14.1	40–60	8
Mm. adductors	83.3	36.7	20–120	24
M. quadriceps femoris	63.3	29.4	40–120	24
Hamstrings	65.0	25.2	40–100	16
M. gastrocnemius/Caput mediale	56.5	20.3	40–120	68
M. gastrocnemius/Caput laterale	52.7	10.1	40–60	44
M. soleus	53.3	19.7	40–100	48
M. tibialis posterior	71.8	25.6	40–120	68
M. tibialis anterior	50.0	14.1	40–60	8
M. flexor digitorum brevis	88.0	47.3	40–200	40
M. flexor digitorum longus	60.0	16.3	40–80	16
M. extensor hallucis longus	52.5	21.2	20–80	32
M. flexor hallucis longus	68.6	25.4	40–100	28

Analysis of 25 consecutive patients. (A) Patient characteristics, additional target muscles, and general botulinum toxin therapy characteristics. (B) Target muscles, botulinum toxin doses and target muscle usage

*M ± SD* mean ± standard deviation

Leg spasticity is the third largest group of patients treated for spasticity. In our group of 25 consecutive leg spasticity cases, the patient age was 53.7 ± 14.2 years and the patient sex ratio 32% males and 68% females. The total BT dose in leg muscles was 270.4 ± 95.7 MU (minimum 40 MU and maximum 400 MU). The number of leg target muscles treated was 4.3 ± 1.4 (minimum 1, maximum 7). Most frequently used target muscles were M. gastrocnemius/Caput mediale (68%), M. tibialis posterior (68%), M. soleus (48%) and M. gastrocnemius/Caput laterale (44%). Involvement of M. quadriceps femoris should be treated carefully as its muscle tone secures stance. Treatment of the equinovarus posture provides improvement of stance and bears little risk of adverse effects.

### Paraspasticity (Table 12)

**Table 12:** Dosing tables for paraspasticity

(A) Patient age (*M* ± SD) (years)	Patient sex ratio (%)	Additional target muscles (%)	Number of target muscles (*M* ± SD) (*n*)	Total botulinum toxin dose (*M* ± SD) (MU)
48.3 ± 12.0	Males: 50		Average: 6.8 ± 4.2	Average: 584.5 ± 245.8
	Females: 50		Minimum: 2	Minimum: 200
			Maximum: 15	Maximum: 1100

(B) Target muscle	Botulinum toxin dose			Target muscle usage (% per subtype)
	Typical dose (mean) (MU)	Dose variability (standard deviation) (MU)	Dose limits (min–max) (MU)	
M. iliopsoas	50.0	11.5	40–60	5
Mm. adductors	115.8	66.7	40–200	33
M. quadriceps femoris	60.0	28.3	40–120	15
Hamstrings	156.3	100.4	40–400	25
M. gastrocnemius/Caput mediale	57.3	38.7	20–200	28
M. gastrocnemius/Caput laterale	32.9	12.7	20–60	18
M. soleus	70.0	81.6	20–400	28
M. tibialis posterior	66.7	24.4	40–120	18
M. tibialis anterior	70.0	42.4	40–120	5
M. flexor digitorum brevis	66.7	52.9	40–160	13
M. flexor hallucis longus	50.0	11.5	40–60	5

Analysis of 20 consecutive patients. (A) Patient characteristics, additional target muscles, and general botulinum toxin therapy characteristics. (B) Target muscles, botulinum toxin doses and target muscle usage

*M ± SD* mean ± standard deviation

Paraspasticity is the fourth largest group of patient treated for spasticity. In our group of 20 consecutive paraspasticity cases, the patient age was 48.3 ± 12.0 years and the patient sex ratio 50% males and 50% females. The total BT dose in leg muscles was 584.5 ± 245.8 MU (minimum 200 MU, maximum 1100 MU). The number of leg target muscles treated was 6.8 ± 4.2 (minimum 2, maximum 15). Most frequently used target muscles were Mm. adductores (33%), M. gastrocnemius/Caput mediale (28%), M. soleus (28%) and the hamstrings (25%). If the number of leg muscles involved becomes too large for BT therapy, continuous intrathecal baclofen therapy becomes an alternative (Dressler et al. 2015).

### Tetraspasticity (Table 13)

**Table 13:** Dosing table for tetraspasticity

(A) Patient age (*M* ± SD) (years)	Patient sex ratio (%)	Additional target muscles (%)	Number of target muscles (*M* ± SD) (*n*)	Total botulinum toxin dose (*M* ± SD) (MU)
43.2 ± 16.5	Males: 57	M. masseter: 10	Average: 13.1 ± 5.5	Average: 806.8 ± 342.3
	Females: 43		Minimum: 2	Minimum: 80
			Maximum: 24	Maximum: 1340

(B) Target muscle	Botulinum toxin dose			Target muscle usage (% per subtype)
	Typical dose (mean) (MU)	Dose variability (standard deviation) (MU)	Dose limits (min–max) (MU)	
M. pectoralis	60.5	23.0	40–120	77
Mm. latissimus dorsi/teres mai	60.0	22.8	20–100	50
M. biceps brachii	55.3	20.5	10–100	80
M. brachioradialis	47.5	22.9	20–80	33
M. triceps brachii	40.0	16.3	20–60	10
M. brachialis	32.5	10.4	20–40	17
M. flexor carpi ulnaris	57.2	20.5	40–120	53
M. flexor carpi radialis	61.7	24.8	40–120	30
M. extensor carpi ulnaris	40.0	0	40	7
M. flexor digitorum superficialis	75.2	29.0	40–140	47
M. flexor digitorum profundus	82.5	46.5	40–200	53
M. flexor pollicis longus	30.0	11.5	20–40	13
M. pronator teres	40.0	0	40–40	17
Thumb clench	40.0	0	40–40	27
Mm. lumbricales	75.0	30.0	60–120	10
M. iliopsoas	80.0	18.9	40–100	17
Mm. adductors	95.6	46.8	40–200	33
M. quadriceps femoris	78.3	32.7	40–160	40
Hamstrings	80.0	44.0	40–200	37
M. gastrocnemius/Caput mediale	44.0	16.7	20–100	37
M. gastrocnemius/Caput laterale	42.2	18.6	20–80	17
M. soleus	43.1	7.5	40–60	23
M. tibialis posterior	66.0	18.9	40–80	20
M. tibialis anterior	40.0	0	40	10
M. flexor digitorum brevis	53.3	10.0	40–60	17
M. flexor digitorum longus	50.0	11.5	40–60	7
M. extensor hallucis longus	46.7	10.3	40–60	10

Analysis of 30 consecutive patients. (A) Patient characteristics, additional target muscles, and general botulinum toxin therapy characteristics. (B) Target muscles, botulinum toxin doses and target muscle usage

*M ± SD* mean ± standard deviation

Tetraspasticity is the smallest group of patient treated for spasticity. In our group of 30 consecutive tetraspasticity cases, the patient age was 43.2 ± 16.5 years and the patient sex ratio 57% males and 43% females. The total BT dose in arm and leg muscles was 896.8 ± 342.3 MU (minimum 80 MU and maximum 1340 MU). The number of arm and leg target muscles treated was 13.1 ± 5.5 (minimum 2, maximum 24). Most frequently used target muscles were M. biceps brachii (80%), M. pectoralis (77%), M. flexor carpi ulnaris (53%), M. flexor digitorum profundus (53%) and M. flexor digitorum superficialis (47). Additional continuous intrathecal baclofen therapy may become an option, especially to boost efficacy in the legs (Dressler et al. 2015).
